# Involvement of Flavonoids from the Leaves of* Carya cathayensis *Sarg. in Sirtuin 1 Expression in HUVEC Senescence

**DOI:** 10.1155/2018/8246560

**Published:** 2018-07-08

**Authors:** Yan Guo, Liwan Xing, Chaodong Qian, Zhishan Ding, Bo Jin

**Affiliations:** ^1^College of Life Science, Zhejiang Chinese Medical University, Hangzhou, Zhejiang 310053, China; ^2^College of Medical Technology, Zhejiang Chinese Medical University, Hangzhou, Zhejiang 310053, China

## Abstract

Atherosclerosis is the commonest cause of death in the world and one of the most important processes that occurs with increasing age because it is accompanied by progressive endothelial dysfunction. Recent studies demonstrated that Sirtuin 1 (SIRT1) might potentially affect cell senescence. However, the effect of SIRT1 on the regulation of human umbilical vein endothelial cell (HUVEC) senescence with total flavonoids (TFs) has not been addressed previously. This study investigated how SIRT1 functions in the process of HUVEC senescence when TFs are present and identified the potential molecular mechanisms involved. Using a model of HUVEC senescence induced by angiotensin II, TFs pretreatment reduced the percentage of senescence-associated *β*-galactosidase (SA-*β*-gal) cells and p53 mRNA expression. The level of SIRT1 protein and E2F1 decreased during HUVEC senescence and could be partially recovered when cells were coincubated with TFs, while the levels of proteins p53 and p21 increased during cell senescence and diminished in response to the TFs treatment. When coincubated with 20 mM nicotinamide, the results with SA-*β*-gal-positive cells and the expression of SIRT1, E2F1, p53, and p21 were contrary to that obtained with only TFs pretreatment. The data indicate that the TFs exert their effect on HUVEC senescence through SIRT1.

## 1. Introduction

Atherosclerosis is the most widespread cause of death in the UK and is considered to be the most common cause of death in the world [1]. There are many reasons for the occurrence of atherosclerosis, and one of the most important factors is increasing age, which is accompanied by progressive endothelial dysfunction [2]. The incidence, prevalence, and mortality of atherosclerosis increase with age [1], and cellular senescence, genomic instability, and telomere attrition also occur with increasing age [1]. Cellular senescence refers to the state of permanent cell cycle arrest when cells respond to exogenous and endogenous stress signals [3], including replicative senescence (RS) [4] and stress-induced premature senescence (SIPS) [5], which is characterized by a lack of proliferative activity and DNA damage markers [6]. Different cells have different cellular senescence characteristics and biomarkers, such as increased senescence-associated beta-galactosidase (SA-*β*-gal) activity, cell cycle arrest [7], and increased p21 and p53 activity [8]. The tumor suppressor gene, p53, is involved in many aspects of cell biology, including cell proliferation, senescence, and death [8].

Sirtuin 1 (SIRT1) is an NAD-dependent deacetylase and is highly expressed in endothelial cells [9]. SIRT1 regulates the senescence secretome components and apoptosis in response to oxidative and genotoxic stress, as well as age-related diseases [10, 11]. It plays key roles in cell senescence, lifespan extension [12, 13], and stress modulation, which act against aging and age-related diseases through p53 deacetylation. p53 can be regulated by posttranslational modifications, such as acetylation and phosphorylation in addition to regulation at the mRNA and protein levels. p21 is a downstream gene of p53, activated by activated p53. Langley E proved that the promyelocytic leukemia (PML) protein can activate p53 by posttranslational acetylation, while SIRT1 deacetylated p53 to retard PML-induced cell senescence [14]. E2F1, as a cell cycle and apoptosis regulatory factor, can induce cell cycle arrest and apoptosis by a variety of mechanisms of p53-dependent and p53-independent pathways. E2F1 is also an important substrate of SIRT1, which can induce the expression of SIRT1 [15].

Our previous studies have demonstrated that the total flavonoids (TFs) from the leaves of* Carya cathayensis *Sarg. inhibited endothelial cell senescence induced by angiotensin II (Ang II) [16]. This study investigated how SIRT1 functions in the process of HUVEC senescence when TFs are present and identified the potential molecular mechanisms involved.

## 2. Materials and Methods

### 2.1. Materials

The leaves of* Carya cathayensis *Sarg. collected in this experiment were identified by professor Zhishan Ding of Zhejiang Chinese Medical University. The total flavonoids (TFs) were isolated from the leaves of* Carya cathayensis *Sarg. A voucher specimen used in this study has been deposited in molecular biology laboratory of Zhejiang Chinese Medical University (No. LCC-20160915-G).

### 2.2. Cell Culture

The human umbilical vein endothelial cells (HUVECs) were purchased from Institute of Biology (Chinese Academy of Sciences). The cells were cultured in RPMI 1640 medium (Gino, Hang Zhou, China), containing 10% heat-inactivated fetal bovine serum (FBS, Tianhang) at 37°C in a 95% O_2_ and 5% CO_2_ incubator. Cells at passages 3 to 8 were used for the experiment. To establish the aging model, HUVECs were induced by Ang II (10^−6^mol/L) for 48h [17]. For the studies on SIRT1 gene expression, cells were cultured in medium supplemented with nicotinamide (NAM) (Biyuntian, China) for 24 h.

### 2.3. Senescence-Associated *β*-Galactosidase Activity

The SA-*β*-gal staining was performed according to the manufacturer's instructions (Beyotime, China). Briefly, after being treated with fixation fluid for 15 min, the cells were incubated with staining solution overnight at 37°C without CO_2_. The positive cells characteristically display a perinuclear precipitation of blue dye, which allows for clear identification with standard light microscopy. The percentage of SA-*β*-gal-positive cells was observed after a count of a total of 500 cells per culture dish.

### 2.4. Isolation of RNA and Real-Time RT-PCR

Total RNA was extracted with TRIzol reagent. The complementary DNA was synthesized using Prime Script™ RT Master Mix (Perfect Real Time) (Takara, Dalian, China) for real-time polymerase chain reaction (RT-PCR) with conditions of 37°C for 15 min and 85°C for 5 sec and storage at 4°C. The RT-PCR was performed in duplicate using SYBR®Premix Ex Taq™ II (Tli RNaseH plus) (Takara, Dalian, China) at 95°C for 2 min and then 95°C for 5 s, 56°C for 30 s, and 72°C for 30 s for 40 cycles. The primer sequences used in this study were as follows: SIRT1 forward primer: ACTTCAGGTCAAGGGATG; reverse primer: CACTGCACAGGCACATAC; p53 forward primer: GTCTACCTCCCGCCATAA; reverse primer: CATCTCCCAAACATCCCT; p21 forward primer: TTGCGATGCGCTCATGGCGA; reverse primer: CCAGTGGCGTCTCAGTGGCG; E2F1 forward primer: GTTTCCAGAGATGCTCACCTTGTC; reverse primer: ACACCACACAGACTCCTTCCCTT.

### 2.5. Western Blot (WB) Analysis

Briefly, proteins were extracted with radio immunoprecipitation assay (RIPA) solution, and the concentration was measured with a bicinchoninic acid (BCA) kit (Beyotime, China). Proteins samples were separated by SDS-PAGE using a 10% polyacrylamide gel. Then, membranes were exposed to anti-SIRT1 (1:5000 dilution), anti-p21 (1:2000 dilution), anti-p53 (1:2000 dilution), anti-E2F1 (1:5000 dilution), and anti-*β*-actin (1:5000 dilution) overnight at 4°C. The membranes were washed (three times, 10 minutes each) in Tris-buffered saline (TBS) containing 0.1% Tween-20 (TBST) and then incubated with the corresponding secondary antibody.

### 2.6. Statistics

All data are presented as the mean ± SD. Statistical analysis was performed with one-way ANOVA and Dunnett's post hoc test. P values less than 0.05 were considered to indicate statistical significance.

## 3. Results

**Figure 1:**
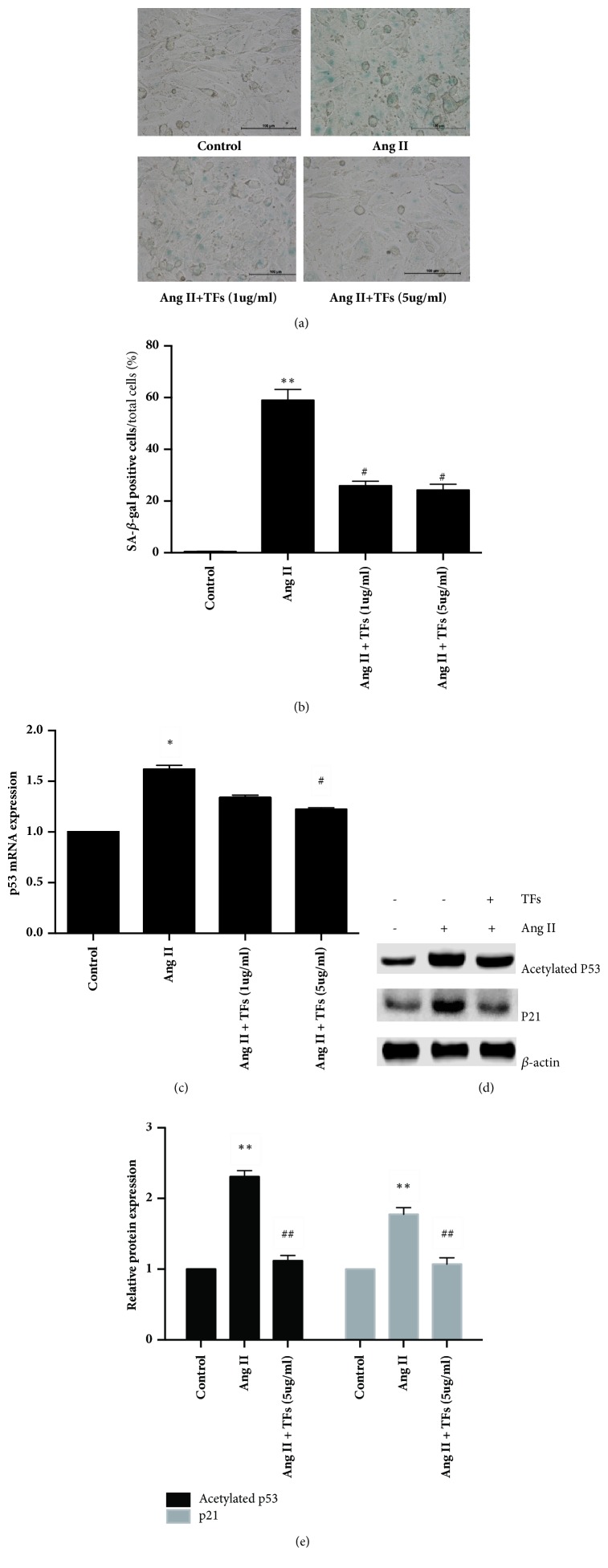
**TFs could inhibit HUVECs senescence.** Panels** (a and b)**: representative images of SA-*β*-gal staining and quantification of SA-*β*-gal positive cells (blue staining for the senescent cell). Scale bar = 100 *μ*m. Panel** (c)**: quantitative analysis of p53 in HUVECs. Panels** (d and e)**: representative images of WB analysis and the semiquantification of p53 or p21 in HUVECs. Values in panels** (b, c, and e)** are expressed as mean ± SD (n = 3). *∗*p < 0.01 and *∗∗*p < 0.01 versus control; ^#^p < 0.05 and ^##^p < 0.01 versus Ang II.

Senescence-associated beta-galactosidase (SA-*β*-gal) activity and p53 and p21 expression are cellular senescence characteristics. As shown in Figures 1(a)–1(c), TFs pretreatment reduced the percentage of SA-*β*-gal-positive cells and p53 mRNA expression, with the 5 *μ*g/ml dose of TFs being more effective. In order to further demonstrate whether the TFs could inhibit HUVEC senescence, we selected the 5 *μ*g/ml dose of TFs to explore the expression of p53 and p21. The results showed that TFs pretreatment reduced the expression of p53 and p21 protein (Figures 1(d)-1(e)).

**Figure 2:**
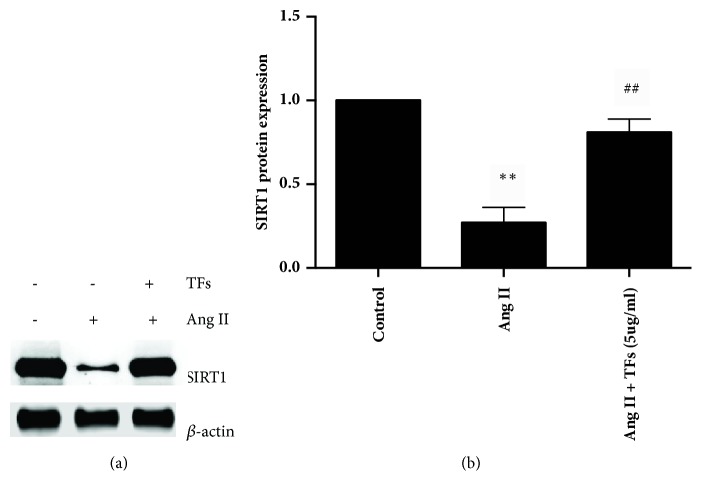
**TFs exert effect on SIRT1 expression.** Panels** (a and b)**: representative images of WB analysis and the semiquantification of SIRT1 in HUVECs. Values in panel** (b)** are expressed as mean ± SD (n = 3). *∗*p < 0.01 and *∗∗*p < 0.01 versus control; ^#^p < 0.05 and ^##^p < 0.01 versus Ang II.

SIRT1 is a known regulator of age-related diseases and is highly expressed in endothelial cells. SIRT1 is slightly expressed in aging cells but its expression increased after TFs treatment. Thus, we demonstrated that TFs affect SIRT1 expression, as shown in Figures 2(a)-2(b).

**Figure 3:**
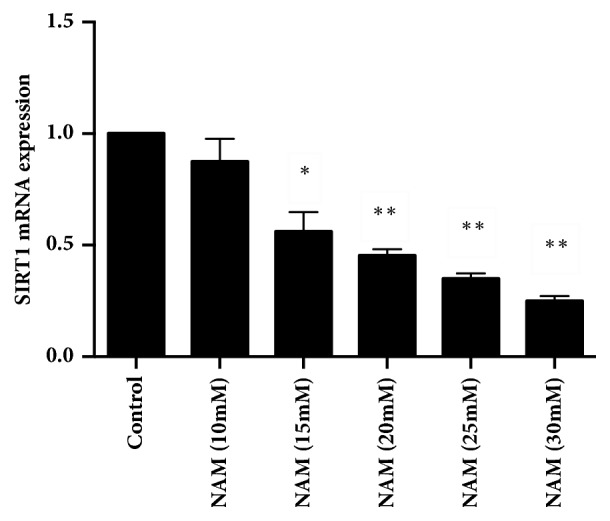
**Optimum concentration of SIRT1.** Quantitative analysis of SIRT1 in HUVECs. Values are expressed as mean ± SD (n = 3). *∗*p < 0.01 and *∗∗*p < 0.01 versus control.

NAM markedly slowed SIRT1 expression in a dose-dependent manner in the range of 10-30 mM in the RT-PCR analysis (Figure 3). NAM at 15 mM resulted in a significant decrease in SIRT1 gene expression compared to the control medium (P < 0.05). NAM at 20 mM showed a significant augmentation in gene expression compared to the control medium and NAM at 15 mM (P < 0.01), in a dose-dependent manner.

**Figure 4:**
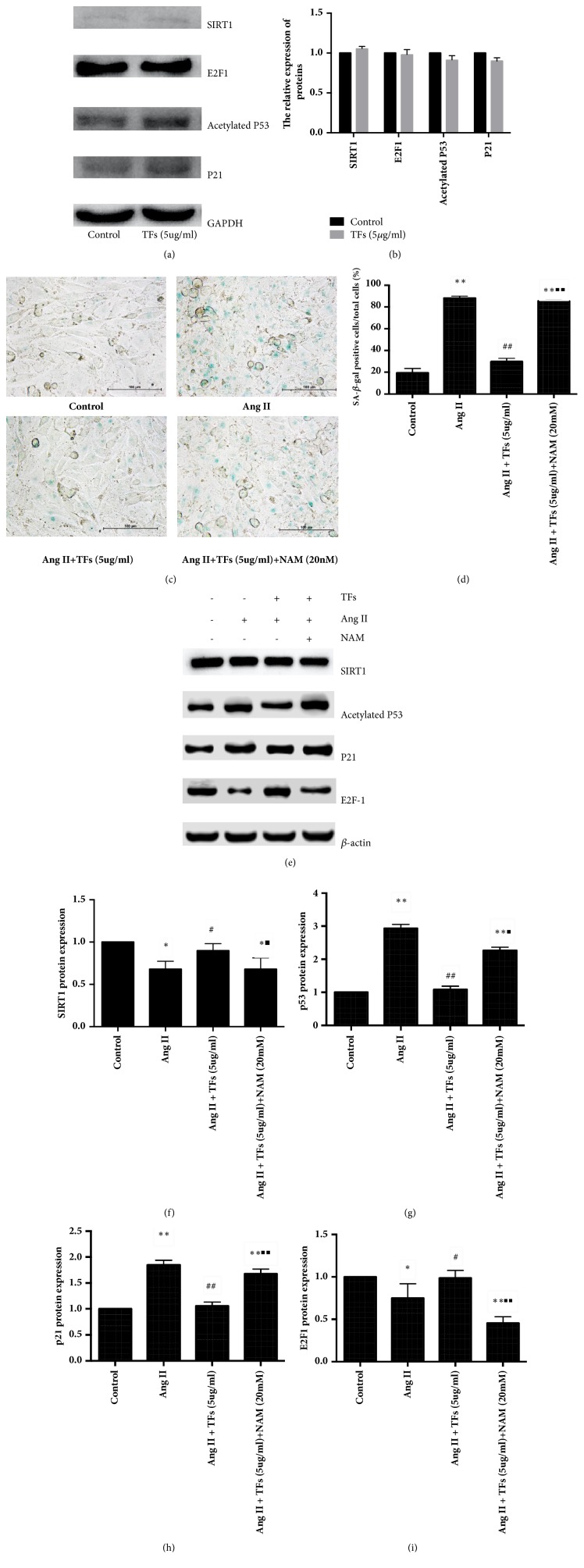
**Effect of SIRT1 on HUVECs senescence regulated by TFs.** Panels** (a and b)**: representative images of WB analysis and the semiquantification of SIRT1, E2F1, p53, and p21 in normal HUVECs. Panels** (c and d)**: representative images of SA-*β*-gal staining and quantification of SA-*β*-gal positive cells (blue staining for the senescent cell). Scale bar = 100 *μ*m. Panels** (e**–**i)**: representative images of WB analysis and the semiquantification of SIRT1, p53, p21, and E2F1 in HUVECs. Values in panel are expressed as mean ± SD (n = 3). *∗∗*p < 0.01 statistically significant difference compared to control. *∗*p < 0.01 and *∗∗*p < 0.01 versus control. ^#^p < 0.05 and ^##^p < 0.01 versus Ang II. ^■^p < 0.01 and ^■■^p < 0.01 versus TFs + Ang II.

Cell senescence was evaluated by SA-*β*-gal staining, and the protein levels of SIRT1, p53, p21, and E2F1 were assessed by western blotting. As shown in Figures 4(c) and 4(d), during in vitro subculture, the percentage of positively SA-*β*-gal-stained cells significantly increased in the senescent cells, whereas the cells preincubated with 5 *μ*g/mL TFs had significantly fewer senescent cells. When coincubated with 20 mM NAM, the number of senescent cells increased. The levels of SIRT1 protein and E2F1 decreased during HUVEC senescence and could be partially recovered when cells were coincubated with TFs, while the levels of proteins p53 and p21 increased during cell senescence and diminished in response to the TFs treatment (Figures 4(e)–4(i)). Meanwhile, we found that TFs took no significant effect on the expression of SIRT1, E2F1, p53, and p21 in normal HUVECs (Figures 4(a)-4(b)).

## 4. Discussion

Senescence-associated beta-galactosidase (SA-*β*-gal) activity and p53 (tumor suppressor gene) are cellular senescence biomarkers [18]. Our experiments showed that the percentage of SA-*β*-gal-positive cells and p53 or p21 expression increased after inducing Ang II, and all these parameters decreased after TF treatment. Above all, we can confirm that the TFs inhibited HUVEC senescence.

SIRT1 is considered to be the most important factor involved in vascular balance, and it modulates a variety of molecular signaling pathways essential for vascular function [9]. SIRT1 is highly expressed in endothelial cells [9, 19]. Endothelial SIRT1 protects vessels against vascular aging, vascular injury, and tissue damage. Our experiments demonstrated that TFs recovered the expression of SIRT1 even with HUVEC senescence.

SIRT1 binds to NAD+ and acetyllysine residues within its protein targets to generate lysine, 2'-O-acetyl-adenosine diphosphate- (ADP-) ribose, and nicotinamide as enzymatic products. Nicotinamide acts as a negative-feedback inhibitor of SIRT1 [20]. When we used NAM, the expression of SIRT1 mRNA decreased in a dose-dependent manner. Although using siRNA or shRNA to inhibit SIRT1 expression may be better, using NAM (20mM) to inhibit sirt1 also has significant results. Future studies we will consider further verification using siRNA or shRNA.

High levels of SIRT1 consume and deplete NAD+, which results in adenosine triphosphate (ATP) deficiency and subsequent cell death, because NAD+ is necessary for mitochondrial respiration [21]. Nicotinamide phosphoribosyltransferase (NAMPT) is a key enzyme that controls the availability of NAD+ for SIRT proteins [22]. A marked decline in NAMPT activity precedes vascular smooth muscle cell (VSMC) replicative senescence, and NAMPT overexpression in aging VSMCs confers resistance to oxidative stress and delays their senescence via enhanced deacetylation of p53 by SIRT1 [23]. Activated p53 further activates downstream gene P21. It has been previously shown that E2F1 and p53 modulate SIRT1 expression under oxidative stress, DNA damage conditions, and nutrient deprivation. E2F1 directly binds to the SIRT1 promoter at a consensus site and appears to regulate the basal expression levels of SIRT1 [24]. SIRT1 deacetylates and inactivates p53 to antagonize RS and SIPS [14, 17, 25–27]. Our experiments showed that when coincubated with 20 mM NAM, the results of SA-*β*-gal-positive cells and the expression of SIRT1, E2F1, p53, and p21 were contrary to that which was obtained with only TFs pretreatment. Moreover, TFs have no effect on related factors in normal cell but only in senescent cells.

## 5. Conclusions

In conclusion, our results show that the TFs inhibited HUVEC senescence and exerted their effect by modulating SIRT1 expression.

## Data Availability

The data used to support the findings of this study are available from the corresponding author upon request.
